# A Multi-Task Group Bi-LSTM Networks Application on Electrocardiogram Classification

**DOI:** 10.1109/JTEHM.2019.2952610

**Published:** 2019-11-12

**Authors:** Qiu-Jie Lv, Hsin-Yi Chen, Wei-Bin Zhong, Ying-Ying Wang, Jing-Yan Song, Sai-Di Guo, Lian-Xin Qi, Calvin Yu-Chian Chen

**Affiliations:** 1 Artificial Intelligence Medical Center, School of Intelligent Systems EngineeringSun Yat-sen University 26469 Shenzhen510275China; 2 School of Software and Applied TechnologyZhengzhou University 12636 Zhengzhou450002China; 3 Department of Medical ResearchChina Medical University Hospital 38020 Taichung40447Taiwan; 4 Department of Bioinformatics and Medical EngineeringAsia University 63267 Taichung41354Taiwan

**Keywords:** ECG, bidirectional long short-term memory network, attention mechanism, multi-task learning

## Abstract

Background: Cardiovascular diseases (CVD) are the leading cause of death globally. Electrocardiogram (ECG) analysis can provide thoroughly assessment for different CVDs efficiently. We propose a multi-task group bidirectional long short-term memory (MTGBi-LSTM) framework to intelligent recognize multiple CVDs based on multi-lead ECG signals. Methods: This model employs a Group Bi-LSTM (GBi-LSTM) and Residual Group Convolutional Neural Network (Res-GCNN) to learn the dual feature representation of ECG space and time series. GBi-LSTM is divided into Global Bi-LSTM and Intra-Group Bi-LSTM, which can learn the features of each ECG lead and the relationship between leads. Then, through attention mechanism, the different lead information of ECG is integrated to make the model to possess the powerful feature discriminability. Through multi-task learning, the model can fully mine the association information between diseases and obtain more accurate diagnostic results. In addition, we propose a dynamic weighted loss function to better quantify the loss to overcome the imbalance between classes. Results: Based on more than 170,000 clinical 12-lead ECG analysis, the MTGBi-LSTM method achieved accuracy, precision, recall and F1 of 88.86%, 90.67%, 94.19% and 92.39%, respectively. The experimental results show that the proposed MTGBi-LSTM method can reliably realize ECG analysis and provide an effective tool for computer-aided diagnosis of CVD.

## Graphical abstract

The Group Bi-LSTM and Residual Group CNN (Res-GCNN) were utilized to build classification model to realize intelligent assistant diagnosis of CVD. The result showed the proposed MTGBi-LSTM method can reliably realize any multi-lead ECG (2-lead or more) analysis and provide an effective tool for computer-aided diagnosis of CVD.

## I. Introduction

Cardiovascular disease is the leading cause of death around the world [1]. The total number of deaths caused by CVD increased from 12.3 million (25.8%) in 1990 to 17.9 million (32.1%) in 2015. Deaths caused by CVD are more prevalent at certain ages and are increasing in most developing countries [2], [3]. It is estimated that up to 90% of CVD may be preventable [4], [5]. Most CVDs can be diagnosed by ECG data. ECG is time-series physiological data. The electrical signal is generated by the sinoatrial node, which passes through the atrioventricular node and Purkinje fibers, and finally reaches the ventricle [6].

The automatic analysis of ECG is of great significance to the early detection and treatment of CVD. With the development of AI, more and more deep learning [7] methods are applied to address medical data [8]–[10], such as feedforward neural network [11]–[14], and the Recurrent Neural Networks (RNN) [15] (Long Short-Term Memory (LSTM) [16], [17], Gate Recurrent Unit (GRU) [18]). However, most of previous studies used the MIT-BIH-AR database [19]–[21], which only has 48 patients. And they only used one-lead ECG data for model training. Since the same person’s ECG data fragments are highly correlated [14], [22], this leads to high accuracy. Once other databases are used or are actually used in clinical practice, the performance of their models will decline dramatically and are not robust.

The multi-lead ECG can provide a full evaluation of the cardiac electrical activity (includes arrhythmias, myocardial infarction, conduction disturbances) [23]. Each lead reflects the electrical signal changes in different parts of the heart. The diagnosis of CVD often needs to be combined with the change of electrical signals in different parts of the heart. This means that each lead signal and correlation among the leads have the same importance for intelligent analysis of ECG.

The accuracy of these methods is not high, and these studies are simply predicting a few types of CVD categories [23], [24]. These methods use the depth of the model in exchange for the performance improvement of the model, without exploring the essential characteristics of the ECG. Previous works based on multi-lead ECG analysis did not pay attention to the correlation among different lead information.

The main contributions of this work can be concluded as follows

1. The number of leads for existing ECG data is not uniform, so we design a Multi-Task Group Bi-LSTM (MTGBi-LSTM), which uses any multi-lead ECG (2-lead or more) learning to simulate the assistant diagnostic process of cardiologists. In the training phase, the model can change the number of its groups according to the number of ECG leads input. This is very promising and influential for the clinical environment.
2. We propose a Group Bi-LSTM module, a combination of Global Bi-LSTM and Intra-Group Bi-LSTM, which can learn each lead individually and fuse the global information of different leads, especially it can sparse autoencoding ECG data. Its effectively enhances the expressiveness of signal representation with respect to ECG waveform features.
3. Last but not least, this is the first time that a multi-task framework has been applied to the CVD-assisted diagnosis. In addition, an attention mechanism for the temporal characteristics of ECG is proposed, which provides discriminative feature representation for multi-task module. And then a dynamic weighted loss function is proposed to solve the unbalanced proportion of positive and abnormal diseases.

## II. Methods

**FIGURE 1.**
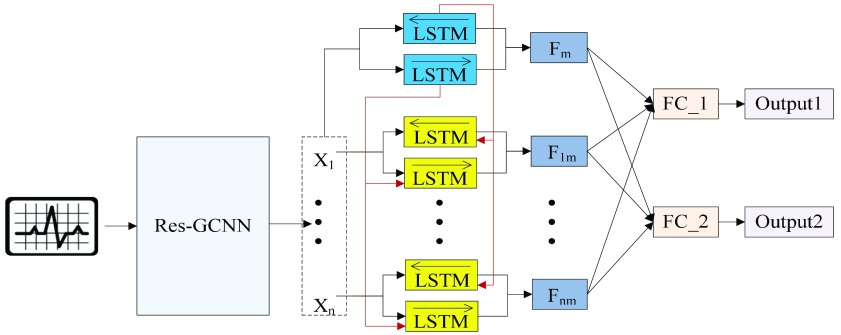
Multi-Task Aided Diagnosis Model Based on Group Bi-LSTM. The Global Bi-LSTM (colored in blue) transmits the hidden state of each time step to Intra-group Bi-LSTM (colored in yellow) through the global access structure (colored in red). $F_{m}$ and $F_{1m} \ldots F_{nm}$ obtained after the output feature of Bi-LSTM passes attention structure, the final fully connected layer (FC_1, FC_2) respectively generate the category distribution of two tasks.

For multi-lead ECGs, we propose a Multi-Task Group Bi-LSTM model framework for deep mining of ECG timing features implicit in each lead and between individual leads. On this basis, attention mechanism is added to enable the model to retain key information with emphasis on the premise of guaranteeing comprehensiveness. In addition, through multi-task learning, the model can fully mine the association information between diseases and obtain more accurate diagnostic results. The model architecture is shown in Fig. 1 and Table 1.

**TABLE 1:** Architectures for MTGBi-LSTM

layer name		Output size		MTGBi-LSTM		
input		$1\times 1900\times 12$				
Res-GCNN	conv1.x	$1\times 1900\times 64$		$1\times 1$, 64, stride $1\times 1$		
		$1\times 950\times 64$		$1\times 3$, 64, stride $1\times 2$		
		$1\times 475\times 256$		$1\times 1$, 256, stride $1\times 2$		
	conv2.x	$1\times 238\times 128$		$1\times 1$, 128, stride $1\times 2$		
		$1\times 119\times 128$		$1\times 3$, 128, stride $1\times 2$		
		$1\times 60\times 512$		$1\times 1$, 512, stride $1\times 2$		
	conv3.x	$1\times 30\times 256$		$1\times 1$, 256, stride $1\times 2$		
		$1\times 15\times 1024$		$1\times 3$, 256, stride $1\times 2\,\,1\times 3$ DC, dilation 2,256, stride $1\times 2\,\,1$x3 DC, dilation 3,256, stride $1\times 2$ avg_pool,1x2, stride $1\times 2$		
		$1\times 8\times 512$		$1\times 1$, 512, stride $1\times 2$		
	avg_pool	$1\times 1\times 512$		$1\times 8$, stride $1\times 1$		
	reshape	$1\times 512$				
Group Bi-LSTM	The Global Bi-LSTM			The Intra-Group Bi-LSTM		
	Global Bi-LSTM	$1\times 512$	256-d	Intra-Group Bi-LSTM	$1\times 512$	256-d
	Attention	$1\times 256$		Attention	$1\times 64$	
	Concat	$1\times 1024$				
Multi-Task Learning	Task 1			Task 2		
	FC_1	$1\times 5$	512-d	FC_2	$1\times 15$	512-d

### A. Residual Group Convolution Neural Network (Res-GCNN)

The different lead data of the ECG is divided into different groups for Res-GCNN, and the number of leads of the ECG is the number of groups that need to be grouped. Such as, the ECG data is input to the top convolution layer in the size of $1\times 1900\times12.\,\,12$ is the number of leads of ECG, this resulted in the number of Res-GCNN being 12.

**FIGURE 2.**
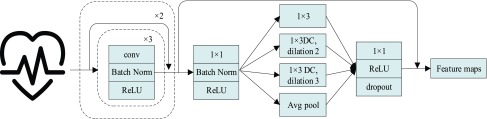
The architecture of the Res-GCNN module. The “X3” in the figure represents three consecutive convolutional layers with kernel sizes of $1\times1$, $1\times3$, and $1\times1$. “X2” represents two consecutive similar residual modules.

The Res-GCNN module of each group is composed of contraction path and expansion path, as show in Fig. 2. And more structural details are in Table 1. In the contraction path, a deep residual network with three residual blocks [25] is designed to encode the single lead data of ECG. The first two residual module has three continuous convolutions layers with kernel size of $1\times1$, $1\times3$, and $1\times1$, and each convolution layer is followed by a rectified linear unit ReLU [26] and Batch Normalization [27] to improve sparsely. In the extended path, the last layer of the last residual block uses three filters of different sizes, which are $1\times3$, $1\times 3$ dilated convolution (1x3DC, dilation rate is set to 2), $1\times 3$ DC (dilation rate: 3), and an avg pool to convolute and pool the input data. The design of Res-GCNN module reduces the network parameters and the thickness of the feature map, and makes the feature map keep more high-order features at different levels, enriching the expressive ability of the network [28]. Finally, all sub-layer outputs within each group are cascaded and transmitted to the Group Bi-LSTM section.

### B. Group Bi-LSTM Module

Group Bi-LSTM is divided into two parts, the global Bi-LSTM and the intra-group Bi-LSTM. The Group Bi-LSTM Module can dynamically change the number of its groups according to the number of ECG leads input, and combine the global Bi-LSTM and intra-group Bi-LSTM autoencoder signal representation to provide high-resolution feature information for the final CVD-assisted diagnosis and recognition.

#### 1) The Global Bi-LSTM

The global Bi-LSTM updates the cell states by combining the long-term state information of the up and down time, after receiving the feature information of all the leads $\sum \limits _{i=1}^{n} {X_{it}} $ at the current time, and synchronously sends the hidden state $h_{gt}$ of the output to the each intra-group Bi-LSTM. There is only one global Bi-LSTM in the Group Bi-LSTM Module.

**FIGURE 3.**
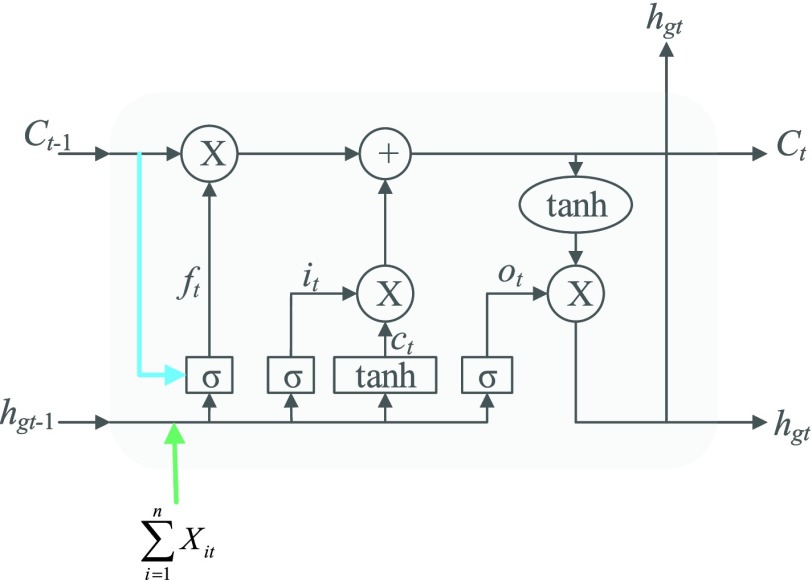
Cell Structure of Global Bi-LSTM. We employed a peephole connections, i.e., the extra blue connections.

In Fig. 3, the global Bi-LSTM removes or adds information to the cell states through three “gates” structure (forget gate, input gate and output gate) [29]. Gate is a way to allow information to pass selectively, including a sigmoid neural network layer and a pointwise multiplication operation. The feature sequence $X=\left ({{X_{11},\ldots,X_{\textrm {i}t}} }\right)\textrm {i}\in \textrm {(1,2,}\ldots,\textrm {n)}$ after Res-GCNN is used as the input of global Bi-LSTM.

The function of forget gate is to selectively discard the feature information of cell state. After inputting the spatial features of the ECG, we hope to pay more attention to the characteristic information of P wave, QRS wave and T wave. In the whole ECG, some relatively flat features may not affect the classification results, so we need to selectively discard them to extract the essential feature information of ECG.

\begin{equation*} f_{t} =\sigma \left ({{W_{f} \left [{ {C_{t-1},h_{\textrm {g}t-1},\sum \limits _{i=1}^{n} {X_{it}}} }\right]+b_{f}} }\right)\tag{1}\end{equation*}

where $X_{\mathrm {it}}$ is the input value in LSTM, that is, the latent discriminative structural feature maps is extracted by the i-th Res-GCNN module. $n$ is the number of leads of ECG. $C_{t-1}$, $h_{gt-1}$ represent the cell state at the previous moment and the output of the previous cell, respectively. $W_{f}$, $b_{f}$ are the parameters to be learned by LSTM. $\sigma $ represents the Sigmoid activation function.

The function of the input gate is to control whether the feature information input at the current time is integrated into the memory unit. When it receives a certain feature of the ECG, it may be important for the prediction of the entire ECG or not.

\begin{align*} i_{t}=&\sigma \left ({{W_{i} \left [{ {h_{\textrm {g}t-1},\sum \limits _{i=1}^{n} {X_{it}}} }\right]+b_{i}} }\right) \tag{2}\\ \widetilde {C}_{t}=&\varphi \left ({{W_{c} \left [{ {h_{\textrm {g}t-1},\sum \limits _{i=1}^{n} {X_{it}}} }\right]+b_{c}} }\right) \tag{3}\\ C_{t}=&f_{t} \odot C_{t-1} +i_{t} \odot \widetilde {C}_{t}\tag{4}\end{align*}

where $W_{i}, W_{c}, b_{i}, b_{c} $ are the parameters to be learned by LSTM. $C_{t}$ represents the state of the cell at the current moment. $\varphi $ represents the *Tanh* activation function. The rest of the symbols have the same meaning as Eq. (1).

The purpose of the output gate $O_{t} $ is to generate a hidden layer unit $h_{gt} $ from the memory unit $C_{t}$. However, not all information in $C_{t}$ is related to the hidden layer unit $h_{gt}$. $C_{t}$ may contain a lot of information that is useless to $h_{gt}$. Therefore, the role of $O_{t}$ is to determine which parts of $C_{t}$ are useful for $h_{gt}$ and which parts are useless.

\begin{align*} O_{t}=&\sigma \left ({{W_{o} \left [{ {h_{\textrm {g}t-1},\sum \limits _{i=1}^{n} {X_{it}}} }\right]+b_{o}} }\right) \tag{5}\\ h_{\textrm {g}t}=&O_{t} \odot \varphi \left ({{C_{t}} }\right)\tag{6}\end{align*}

where W_o_, b_o_ are the parameters to be learned by LSTM. $h_{gt}$ represents the output of the cell at the current moment.

#### 2) The Intra-Group Bi-LSTM

The intra-group Bi-LSTM only accepts the feature map information of a single lead $X_{\mathrm {it}}$. After introducing the inter-lead feature information $h_{gt}$ from the global Bi-LSTM through the global access structure, updates the state of the cell by combining the ECG information at the time before and after. The input of ECG data has *n-* lead, and the Group Bi-LSTM Module has $n$ Intra-Group Bi-LSTM.

**FIGURE 4.**
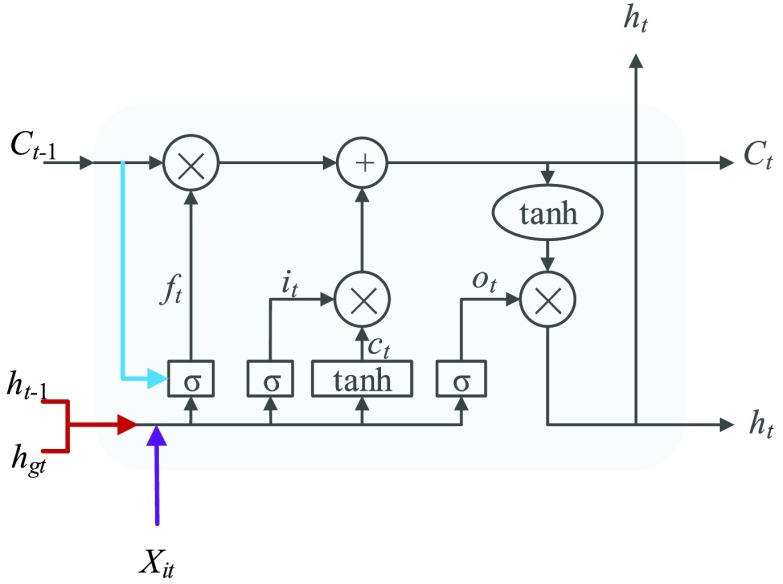
Cell Structure of Intra-group Bi-LSTM. $h_{gt}$ is the inter-lead feature information introduced from the global Bi-LSTM through the global access structure.

In Fig. 4, the intra-group Bi-LSTM removes or adds information to the cell state through three “gate” structures. The forward LSTM in the intra-group Bi-LSTM first accepts the hidden state of the forward LSTM in the global Bi-LSTM at the current time, and then combines with the current input sequence feature data and the hidden state of the previous moment to remove and update the cell state at the current time. Another reverse LSTM is similar.

The intra-group Bi-LSTM network computes a mapping from an input sequence $X=\left ({{X_{\textrm {i}1},\ldots,X_{\textrm {i}t}} }\right)\textrm {i}\in \textrm {(1,2,}\ldots,\textrm {n)}$ to an output sequence $h=\left ({{h_{1},\ldots,h_{t}} }\right)$ by calculating the network unit activations using the following equations iteratively from $t =$ *1* to $t$:

\begin{align*} f_{t}=&\sigma \left ({{W_{f} \left [{ {C_{t-1},h_{t-1},h_{\textrm {g}t},X_{it}} }\right]+b_{f}} }\right) \tag{7}\\ i_{t}=&\sigma \left ({{W_{i} \left [{ {h_{t-1},h_{\textrm {g}t},X_{it}} }\right]+b_{i}} }\right) \tag{8}\\ {C}_{t}=&\varphi \left ({{W_{c} \left [{ {h_{t-1},h_{\textrm {g}t},X_{it}} }\right]+b_{c}} }\right) \tag{9}\\ C_{t}=&f_{t} \odot C_{t-1} +i_{t} \odot {C}_{t} \tag{10}\\ O_{t}=&\sigma \left ({{W_{o} \left [{ {h_{t-1},h_{\textrm {g}t},X_{it}} }\right]+b_{o}} }\right) \tag{11}\\ h_{t}=&O_{t} \odot \varphi \left ({{C_{t}} }\right)\tag{12}\end{align*}

where $X_{it}$ represents the input value in the intra-group Bi-LSTM, in other words, ts the latent discriminative structural feature maps is extracted by the i-th Res-GCNN module. It must be noted that it is distinguished from $\sum \limits _{i=1}^{n} {X_{it}} $, the input of Group Bi-LSTM module, in Eq. (1). $h_{gt}$ represents the hidden state of the forward LSTM in the Global Bi-LSTM at the current time, that is, the output state of the cell in the global Bi-LSTM in Eq. (1). $\sigma $, $\varphi $ represents the Sigmoid and the *Tanh* activation function, respectively.$\odot $ represents the element level product operation. $W_{f}, W_{i}, W_{c}, W_{o}, b_{f}, b_{i}, b_{c}, b_{o}$ are the parameters to be learned by LSTM.

### C. The Attention Mechanism

**FIGURE 5.**
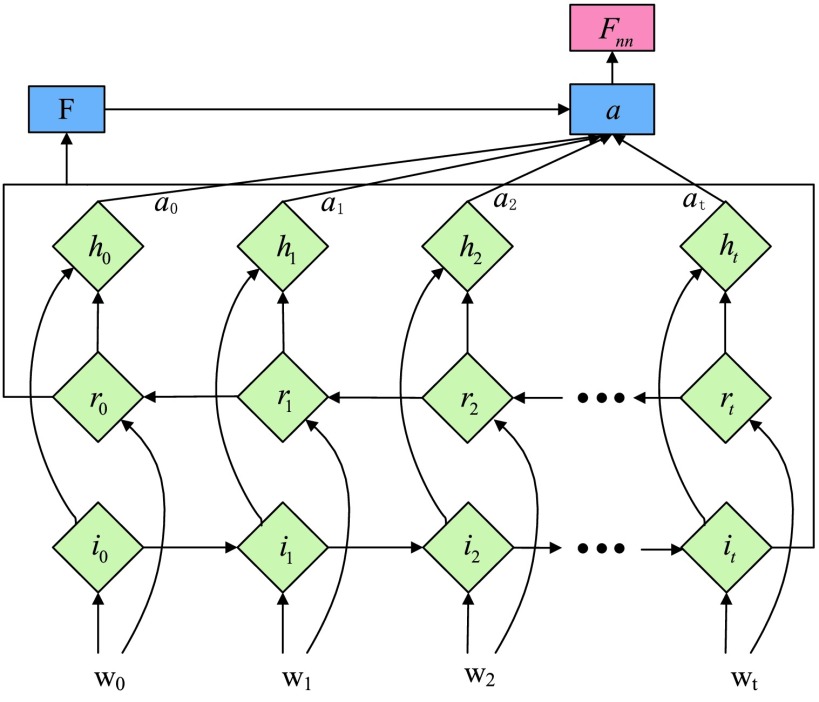
Bi-LSTM Model with Attention Mechanism. $F$ represents the sum of the final hidden layer state values for each independent direction in Bi-LSTM, which is called the final state of Bi-LSTM. $a$ denotes the attention probability distribution of the hidden layer unit state to the final state at all times. $F_{nn}$ denotes the final ECG feature vector weighted by attention in Bi-LSTM.

It must be noted that we employs the attention mechanism [30] on every Bi-LSTM (including the Global Bi-LSTM, Intra-Group Bi-LSTM). The Attention mechanism of this model includes two parts of calculation process. One part is about the process of calculating the probability distribution of attention, the other part is the process of calculating the final feature based on attention distribution (Fig. 5).

$F $ represents the sum of the final hidden layer state values for each independent direction in Bi-LSTM, which is called the final state of Bi-LSTM.$a$ denotes the attention probability distribution of the hidden layer unit state to the final state at all times, and component $a_{t}$ denotes the attention probability of Bi-LSTM state $h_{t} $ to the final state at $t$ times. $h_{t}$ is obtained by adding the states of the each independent directions at that time. $F_{nn}$ denotes the ECG feature vector weighted by attention in Bi-LSTM.

1) The attention probability of the output data for the final state at the moment $t$. Enter the hidden state of the Bi-LSTM network layer at each moment into the attention module to generate a weight vector. The weight matrix is got by the following formula:
  \begin{align*} h_{t}^{\prime }=&h_{t}^{T} UF \tag{13}\\ a_{\textrm {t}}=&\frac {exp \left ({{h_{t}^{\prime }} }\right)}{\sum \limits _{i=1}^{N} {exp \left ({{h_{i}^{\prime }} }\right)}}\tag{14}\end{align*}
  The formula uses the softmax function as the calculation method of attention probability distribution. $N$ denotes the number of input sequence elements. $U$ is the weight matrix. $F$ represents the sum of the final hidden layer state values for each independent direction in Bi-LSTM.$h_{t}$ represents the sum of two-way hidden layer state values at the moment $t$.
2) $F_{nn}$, the final feature based on attention distribution, is expressed as:
  \begin{equation*} F_{nn} =\sum \limits _{n=1}^{N} {a_{t} h_{t}}~ F_{nn} =\sum \limits _{n=1}^{N} {a_{t} h_{t}}\tag{15}\end{equation*}

$N$ denotes the number of input sequence elements. $a_{t}$ represents the attention probability of the output data for the final state at the moment $t$. $\mathrm {h}_{t}$ represents the sum of hidden layer states in two independent directions at the moment $t$.

### D. Dynamic Weighted Loss

Considering that in the field of ECG research, since most subjects have normal heartbeats and a small proportion of lesions, the ECG dataset is generally unbalanced. As shown in the distribution of ECGs in Table 3, N class accounted for 61.10% of the complete data set, SA, AA accounted for 18.14% and 7.72%, respectively, while the remaining 12 categories accounted for only 13.04% of the total. Therefore, there is an urgent need for a robust and stable classification method that can cope with different data sets.

**TABLE 2:** Data Distribution of Different Disease Types

Task1	Task2	Number of Records
Normal Rhythm(Normal)	Normal Rhythm	108830
Arrhythmia(AR)	Sinus Arrhythmia(SA)	32306
	Atrial Arrhythmia(AA)	13742
	Junctional Arrhythmia(JA)	404
	Ventricular Arrhythmia(VA)	3883
	Heart Block(HB)	9131
Myocardial Infarction(MI)	Anterior Myocardial Infarction(AMI)	132
	Inferior Myocardial Infarction(IMI)	320
	Lateral Myocardial Infarction(LMI)	112
Ventricular Hypertrophy(VH)	Left Ventricular Hypertrophy(LVH)	8531
	Right Ventricular Hypertrophy(RVH)	115
	Bilateral Ventricular Hypertrophy(BVH)	93
Atrial Hypertrophy(AH)	Left Atrial Hypertrophy(LAH)	210
	Right Atrial Hypertrophy(RAH)	216
	Bilateral Atrial Hypertrophy(BAH)	84
Total		178109

**TABLE 3:** Data Distribution of Disease Types in Task 1

Task1	Train Set	Val Set	Test Set	Total
Normal	87064	10883	10883	108830
AR	47570	5946	5950	59466
MI	451	56	57	564
VH	6989	875	875	8739
AH	407	51	52	510
Total	142481	17811	17817	178109

In order to solve the problem of this unbalanced data set, most researchers use data augmentation techniques to expand the size of data sets [31]. However, this may take more training time, and for the examples that are difficult to classify (hard examples), the loss corresponding to the training will be very small, resulting in a small gradient change in the inverse calculation, and the convergence of the parameters is limited. Compared with other examples, we need to make this sample produce relatively large loss values.

Our method uses raw input data without adding any extra data. To increase the loss of the hard example, we adjusted the weights of the hard example and easy example. We recommend using the new dynamic weighted loss function defined below. First, we define the set of $M$ heartbeat labels in the $i$-th batch as $B_{i}$, and the $j$-th label in $B_{i}$ as $Y_{i,j}$.

\begin{equation*} B_{i}=\{Y_{i,1}, Y_{i,2},Y_{i,3}, \ldots,Y_{i,M}\}\mathrm {a}\tag{16}\end{equation*}

where $Y_{i,j}$ represents different categories in different tasks, $\text{Y}_{\mathrm {i,j}} \in $ {N, AR, MI, VH, AH} in task one. $\text{Y}_{\mathrm {i,j}} \in $ {N, SA, AA, JA, VA, HB, AMI, IMI, LMI, LVH, RVH, BVH, LAH, RAH, BAH} in task two. Then, the loss weight of the $k$-th class in the $i$-th batch, $c_{i,k}$ is calculated as follows:

\begin{equation*} c_{i,k}=1-\frac {\sum \nolimits _{j=1}^{M} Y_{i,j=k}}{M}+\xi\tag{17}\end{equation*}

Among them, $M$ is the batch size and $\xi $ is set to 0.01 to prevent the loss weight equal to 0 when $B_{i}$ contains only one class. Finally, the weighted cross-entropy loss function of the first batch is calculated, as shown in formula (18).

\begin{equation*} L_{i}= -\sum \nolimits _{j=1}^{M} c_{i,k} Y_{i,j}log\tilde {Y}_{i,j}+{\lambda \left \|{ W }\right \|}_{2}^{2}\tag{18}\end{equation*}

where $\tilde {Y}_{i,j}$ are the predictive probability of the $j$-th training instance in the first batch, $W$ is the weight matrix of all layers, and $\lambda $ is the L2 regularization parameter, which is set to 0.01 in our experiment.

### E. Multi-Task Learning

The single-task model focuses on a single task, ignoring other information that may help optimize metrics among diseases. The module trains two tasks related to ECG prediction in parallel, and the feature representation of one task can also be used by other tasks. It promotes the two ECG tasks to learn together. By sharing the network structure before the multi-task module [32], two tasks are trained in parallel using the shared representation. Each task keeps the relevant output on its own path. This resulted the parameters of ECG classification model are reduced, the phenomenon of over-fitting is avoided to some extent, and the generalization ability of the model is improved.

Combining the feature vectors learned from the group Bi-LSTM module. The final fully connected layer respectively generate the category distribution in each time step of two tasks, and at the same time get the loss function of the two tasks. Combining these two loss values according to different weights, the Adam optimizer algorithm is responsible for minimizing the joint loss value. The final classification loss is as follows:

\begin{equation*} L^{total}=\lambda L^{task1}+\left ({1-\lambda }\right)L^{task2}\tag{19}\end{equation*}

where $\lambda $ is a parameter that interpolates between the task1 and task2 losses, $L^{task1}$, $L^{task1}$ represent the loss functions of task 1 and task 2, respectively.

### F. Dataset

The Chinese Cardiovascular Disease Database (CCDD), the largest ECG database all over the world, is developed by the Chinese Academy of Sciences [33]. More than 18,000 recordings in this database are collected from real world with high quality, each ECG record corresponds to one patient, which has been labeled by professional doctors. The records in the database are all 12-lead ECGs, each with a duration of about 10s, digitized at a rate of 500 sampling points per second. More details of screening patient records were contributed in supplementary information 3. Table 2 shows the data distribution of the ECG data sets after screening.

Each ECG has two different label values, which are used as the label values of two tasks. Task1 was labeled as arrhythmia, myocardial infarction, ventricular hypertrophy, atrial hypertrophy and normal rhythm. Each ECG tag was labeled as normal rhythm and 14 subtypes of diseases corresponding to the four diseases in Task 1, including sinus arrhythmia, heart block, anterior myocardial infarction, left ventricular hypertrophy, etc. These 15 subtypes were labeled as Task2 label. The specific ECG distribution is shown in Table 3 and Table 4.

**TABLE 4:** Data Distribution of Disease Types in Task 2

Task2	Train Set	Val Set	Test Set	Total
Normal	87064	10883	10883	108830
SA	25844	3231	3231	32306
AA	10993	1374	1375	13742
JA	323	40	41	404
VA	3106	388	389	3883
HB	7304	913	914	9131
AMI	105	13	14	132
IMI	256	32	32	320
LMI	90	11	11	112
LVH	6824	853	854	8531
RVH	91	12	12	115
BVH	74	10	9	93
LAH	168	21	21	210
RAH	172	22	22	216
BAH	67	8	9	84
Total	142481	17811	17817	178109

## III. Results and Discussion

In this section, we will present the results of the above experiments to verify the effectiveness of MTGBi-LSTM in multi-lead ECG classification tasks and its advantages over existing CNN-based or RNN-based methods.

### A. Performance of MTGBi-LSTM

**FIGURE 6.**
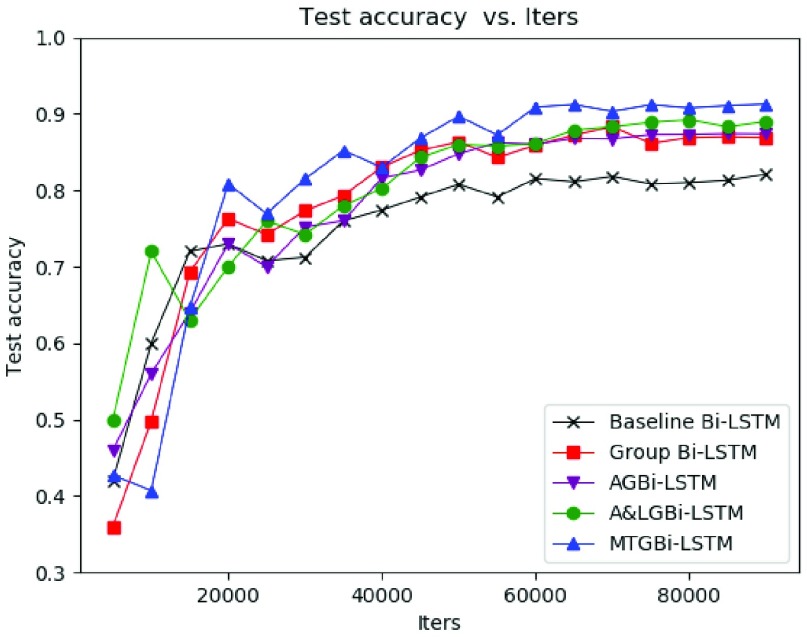
Validation performance graph of comparison networks.

The model training process was evaluated by using the validation set. The classification accuracy of ECG validation set has been basically smooth and stable after 60000 iterations (Fig. 6). The model has stronger information extraction and fitting ability and its performance approximates the optimal.

**FIGURE 7.**
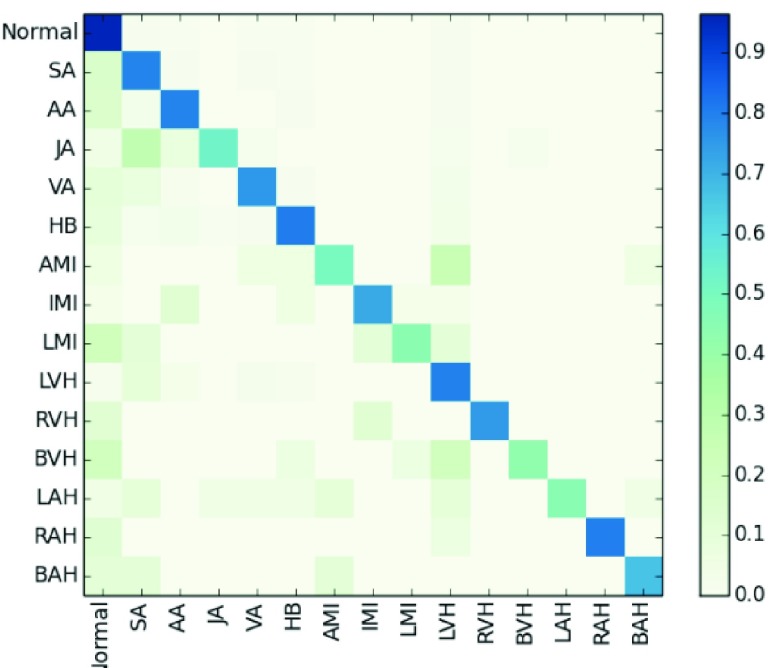
Normalized confusion matrix of model results as percentages for the 15-classproblems. The percentage of all possible records in each category is displayed on a color gradient scale.

The results in Table 5 show that the accuracy, precision, recall, and F1 of MTGBi-LSTM reached the best scores of 0.8886, 0.9067, 0.9419, and 0.9239, respectively. Fig. 7 shows a confusion matrix for the classification results of the MTGBi-LSTM model on the clinical ECG test set. The normal and 14 abnormal rhythms in ECG data set are classified and the best recognition results are obtained. This comparative experiment proves the validity of the MTGBi-LSTM framework proposed by us, which can deeply mine the essential features of ECG and accurately predict it.

**TABLE 5:** Experimental Results of Various Models

Class	Baseline Bi-LSTM	Group Bi-LSTM	AGBi-LSTM	A&LGBi-LSTM	MTGBi-LSTM
Normal	0. 8709	0.9106	0.9297	0.9314	0.9354
SA	0. 7356	0.8329	0.8261	0.8289	0.8458
AA	0. 6053	0.7502	0.8209	0.8272	0.8263
JA	0. 5333	0.4706	0.5052	0.5361	0.5869
VA	0. 5244	0.6024	0.6355	0.6793	0.6918
HB	0. 6513	0.7865	0.7825	0.7965	0.8462
AMI	0. 3846	0.5517	0.4545	0.5218	0.5333
IMI	0 3517	0.4118	0.6572	0.75	0.7188
LMI	0. 3333	0.5	0.5263	0.375	0.4210
LVH	0. 6017	0.6524	0.7137	0.7522	0.7796
RVH	0. 5555	0.6316	0.5883	0.6667	0.6316
BVH	0. 3636	0.56	0.5556	0.5	0.5
LAH	0. 2727	0.4889	0.4	0.5238	0.4390
RAH	0. 4000	0.5263	0.6829	0.6154	0.6487
BAH	0.5000	0.5882	0.5883	0.6667	0.6252
Aggregate Results					
Accuracy	0.7841	0.8497	0.8721	0.878	0.8886
Precision	0.8252	0.8720	0.8965	0.9005	0.9067
Recall	0.8724	0.9224	0.9245	0.9307	0.9419
F1	0.8481	0.8965	0.9103	0.9154	0.9239

The results obtained from the MTGBi-LSTM model are analyzed in detail. As shown in Table 5, the recognition rate of 14 abnormal diseases in the test set reached 85.92%. Among them, the recognition rate of arrhythmia was 87.95%, and the rate of being misclassified as normal category was 4.44%. The model can screen arrhythmias effectively.

The classification and recognition rate of ventricular hypertrophy was 75.77%. The diagnostic rate of being misclassified as normal category was 9.6%. The experimental results show that the model can detect the status of organic lesions in time and warn the subjects to do further examination to find out the potential causes.

ECG data of myocardial infarction and atrial hypertrophy are too little and the data distribution is extremely unbalanced. For both types of identification, the total recall reached 85.92%, only 7 abnormal records are considered normal by the MTGBi-LSTM model. It urges the subjects to do more detailed examinations in order to determine whether they have cardiovascular disease and has early warning effect.

In many cases, the location of the disease is ambiguous and the abnormal rhythm is very similar. Because of the lack of information about subjects, limited signal duration, cardiologists cannot draw reasonable conclusions from ECG. Similar factors also exist in the analysis of ECG which is partially misclassified.

### B. Ablation Study of Framework Component

In order to intuitively show the contribution of the different module to the classification performance, we compared various Bi-LSTM variant models. Baseline Bi-LSTM is a combination of Res-GCNN and common Bi-LSTM. The Group Bi-LSTM is based on the Base Bi-LSTM structure and replaces the common Bi-LSTM with the Group Bi-LSTM module. AGBi-LSTM adds attention mechanism based on Group Bi-LSTM. A&LG-LSTM adds dynamic weighted loss based on AGBi-LSTM, and MTGBi-LSTM adds multi-task learning based on A&LGBi-LSTM. In Table 5, the F1 score that are evaluated under multiple comparative case studies with same testing ECG sample data.

#### 1) Group Bi-LSTM Improves Prediction

The results show that the performance of Group Bi-LSTM is better than that of Baseline Bi-LSTM, in specific, precision, recall and F1 score of Group Bi-LSTM group increased to 0.8720, 0.9224 and 0.8965, respectively. The performance of Group Bi-LSTM is more excels and is a powerful model of feature learning.

The design of Group Bi-LSTM enables the model to deeply exploit the features of single lead and the relationship between each lead of ECG. This means that there is also information flow in the ECG between different leads at the same time. its effectively enhances the quality of feature expression between different ECG leads and the expressiveness of signal representation with respect to ECG waveform features, thus leading to accurate and reliable estimations for CVD. This really is something worth celebrating, a milestone.

#### 2) Attention Mechanism is Useful

The performance of AGBi-LSTM is better than that of Group Bi-LSTM. This shows that the attention mechanism and dynamic weighted loss increases the classification performance. By multiplying the weight vectors in attention mechanism, the influence weights for local temporal features are generated, which can highlight the main waveform features of ECG, and merge the ECG temporal features of each iteration as the main waveform features. Attention probability distribution is used to control the influence of elements in input sequence on elements in output sequence. While retaining more valuable information, reducing the impact of irrelevant or weak correlation information on output data.

The MTGBi-LSTM model combines attention mechanism to selectively learn the final feature map, breaking the limitation of the traditional neural network structure relying on the internal fixed length vector in encoding and decoding, and enhancing the robustness of the model.

#### 3) Dynamic Weighted Loss Has Significant Effect

The results in Table 5 show that the classification performance of the MTGBi-LSTM model has been significantly improved. The F1 values of JA, VA, and LVH have increased by about 4 points. AMI, IMI, RVH, LAH, and, BAH have increased by about 10 points. In particular, the F1 value of LAH increased from 0.4 to 0.5238.

At the same time, we also noticed that the F1 values of LMI, BVH, and RAH have dropped, and there are only about 10 data in the test data set. Manually checking for inconsistencies found that the MTGBi-LSTM classification error overall seemed very reasonable. In some cases, because ECG data records are turbulent, it is not possible to draw reasonable conclusions from the ECG data.

We find that the results are improved when using dynamic weighted loss. It is proved that the dynamic weighted loss function solves the problem of the imbalance of normal and abnormal diseases in the field of intelligent assisted diagnosis.

**Multi-Task learning mechanism is important.** For the symbol $\lambda $ of the multitasking learning mechanism, we evaluate the four values of $\lambda $ to verify the classification accuracy on the test set. As $\lambda $ decreases, the prediction accuracy increases gradually, and all models trained using multitasking learning mechanisms are superior to those that are not trained (Table 6). Because it provides the best prediction accuracy on the test set, in our complete framework, choose $\lambda =0.2$ as the training model.

**TABLE 6:** Evaluation of the Number of ECG Leads and Multi-Task Loss Weight, on the Test Set

	Accuracy	Precision	Recall	F1
$L^{num}=3$,$\lambda =0.2$	0.7893	0.7922	0.8046	0.7984
$L^{num}=8$, $\lambda =0.2$	0.8324	0.8451	0.8542	0.8496
$L^{num}=12$, $\lambda =0.5$	0. 7806	0.8058	0.8456	0.8252
$L^{num}=12$, $\lambda =0.33$	0.8029	0.8303	0.8692	0.8493
$L^{num}=12$, $\lambda =0.2$	0.8886	0.9067	0.9419	0.9239
$L^{num}=12$, $\lambda =0$	0.878	0.9005	0.9307	0.9154

$\lambda $ is a parameter that interpolates between the task1 and task2 losses, $L^{num}$ is the number of leads of ECG data.

The local minimum of different tasks may be located at the low point of different local concave planes. The task1 increases the inter-CVD variations by drawing feature maps extracted from different diseases apart, while the task2 reduces the intra-CVD variations by pulling feature maps extracted from the same disease together. Through different forces between two tasks, the hidden layer can jump out of the local minimum, reduce the risk of model over-fitting, and make the classification accuracy of the model higher.

### C. Effectiveness for Multi-Lead ECG

We evaluate the ECG data of 3-lead, 8-lead, and 12-lead to prove that the MTGBi-LSTM model can intelligently analyze the ECG data of any number of leads. It also demonstrates that the 12-lead ECG can provide a higher quality assessment analysis.

We performed a comparative experiment on the same ECG data set, as shown in Table 3 and Table 4. The labels and the number of records are the same, except that the number of leads used is different. The 3-lead ECG data is selected from I, II, III and the 8-lead ECG data is selected from II, III, V1, V2, V3, V4, V5, V6.

As seen in Table 6, when $L^{num}=3$, $L^{num}=8$ and $L^{num}=12$ ($\lambda = 0.2$) are compared, the precision, Recall, and F1 of the model increase with the number of ECG leads.

The 3-lead based MTGBi-LSTM model predicts both normal and arrhythmia categories, and the predictions for MI, VH, and AH are significantly worse. The ECG information collected by the 3-lead is relatively simple, and the information on the changes of the ST segment and the P wave is not obvious.

The 8-lead based MTGBi-LSTM model clearly achieved better performance than the 3-lead. When $L^{num}=8$, the selected 8-lead are the basic leads of the ECG data, and the remaining four leads of the twelve leads are linearly derived from the eight basic leads. The ECG information collected by the 8 lead is rich, and the MTGBi-LSTM model can extract more highly discriminative ECG essential features.

This shows that our MTGBi-LSTM model can evaluate any multi-lead ECG (2-lead or more) and the 12-lead ECG data based MTGBi-LSTM model achieves the best performance.

### D. Performance Comparison

CNN can stimulate low-dimensional local features implied in ECG waveforms into high-dimensional space, and the subsampling of a merge operation commonly used in CNN can inevitably extract peak points in a given merge window. In previous work [14], [22], [35] (Table VII), the researchers used deep feedforward neural network to classify ECG and achieved high performance.

**TABLE 7:** Comparison of Experimental Results of Different Methods

Author, Year	Database	Number of Lead	ECG Rhythms	Classifier	Acc	Se	Sp	F1
A. H. Ribeiro (2019) [23]	TNMG	12-lead	1dAVb, RBBB,LBBB,SB,AF,ST	CNN	/	0.954	0.996	0.914
Andrew Y. Ng(2019) [14]	91,232 ECG records from 53,549 patients	single-lead	N, AVB, Bigeminy, EAR, IVR, Junctional rhythm, Noise, Sinus rhythm, SVT, Trigeminy, VT, Wenckebach	CNN	/	/	/	0.837
A. Sellami(2019) [35]	MIT-BIH-AR	single-lead	N, S, V, F, Q	CNN	0.9948	0.9697	0.9987	0.9839
X. Zhai(2018) [36]	MIT-BIH-AR	single-lead	N, S, V, F, Q	CNN	0.986	0.938	0.992	0.9642
U. R. Acharya(2017) [37]	afdb,cudb,MIT-BIH-AR	single-lead	Afib, Afl, Vfib, N	CNN	0.949	0.9913	0.8144	0.8942
G. Swapna(2018) [38]	MIT-BIH-AR	single-lead	normal, cardiac arrhythmia	CNN-LSTM	0.834	/	/	/
S. Saadatnejad(2019) [21]	MIT-BIH-AR	single-lead	N, LBBB, RBBB, S, V, F, Q	WT, LSTM	0.986	0.938	0.992	0.9642
A. Mostayed(2019) [24]	CPSCD	12-lead	N, AF, I-AVB, LBBB, RBBB, PAC, PVC, STD, STE	LSTM	/	/	/	0.7415
O. Yildirim(2019) [39]	MIT-BIH-AR	single-lead	APB, LBBB, N, RBBB, PVC	LSTM	0.9911	0.99	0.99	0.99
B. Hou(2019) [40]	MIT-BIH-AR	single-lead	N, LBBB, RBBB, APC, PVC	LSTM,SVM	0.9974	0.9935	0.9984	0.9959
F. Liu(2019) [41]	MIT-BIH-AR	single-lead	N, S, V, F, Q	DWT,LSTM,CNN	0.995	0.999	0.982	0.9904
A. Elola(2019) [42]	OHCA	single-lead	Pulsed Rhythm, Pulseless Rhythm	Bi-GRU	/	0.917	0.925	/
Wang LP [43]	CCDD	12-lead	normal and abnormal	MTHC	0.7770	0.9513	0.5462	/
Zhu HH [44]	CCDD	12-lead	normal and abnormal	multi-classifier	0.7251	0.6198	0.9359	/
Jin LP [25]	CCDD	8-lead	normal and abnormal	LCNN	0.8366	0.8384	0.8343	/
Li HH [45]	CCDD	8-lead	normal and abnormal	CNN	0.8477	0.8519	0.8445	/

Abbreviations: N, normal; AVB, atrioventricular block; EAR, ectopic atrial rhythm; IVR, idioventricular rhythm; SVT, supraventricular tachycardia; VT, ventricular tachycardia; 1dAVb, 1st degree AV block; RBBB, right bundle branch block; LBBB, left bundle branch block; SB, sinus bradycardia; AF, atrial fibrillation; ST, sinus tachycardia; Afib, Atrial fibrillation; Afl, Atrial flutter; Vfib, Ventricular flutter; Vfl, Ventricular flutter; S, supraventricular ectopic beats; V, ventricular ectopic beats; F, fusion beats; Q, unclassifiable beats; PAC, Premature atrial contraction; PVC, Premature ventricular contraction; STD, ST-segment depression; STE, ST-segment elevated; I-AVB, First-degree atrioventricular block; APC, atrial premature complexes Dataset Abbreviations: afdb, MIT-BIH atrial fibrillation, cudb, Creighton university ventricular tachyarrhythmia; MIT-BIH-AR. MIT-BIH arrhythmia; TNMG, Telehealth Network of Minas Gerais; SMCA, Seton Medical Center Austin; CPSCD, the Chinese physiological signal challenge dataset; OHCA, Outof-Hospital Cardiac Arrest database Classifier Abbreviations: DWT, discrete wavelet transform. [45]; LSTM, long short-term memory; CNN, convolutional neural network; WT, wavelet transform; SVM, Support Vector Machine. [46]

As a comparison, results from RNN including those in [21], [23], [41] are listed in Table 7. The advantage of RNN modeling is that the circular network can store a certain length of context information, RNN can handle ECGs of any length of time. When the neural activation unit completes some non-linear mapping, the RNN network can continuously update the network state, so RNN can learn long-term dependencies.

Furthermore, although both the CNN-based method and the RNN-based method obtain good results, those study has several important limitations. The CNN can only learn completely fixed time-varying weights, and it can only accept fixed length ECG input. CNN cannot understand the complex time characteristics in ECG data, ignoring the correlation between ECG data before and after.

The RNN-based algorithm simply superimposes the number of layers of LSTM or GRU, or combines with tricks in deep learning to improve model performance. These algorithms use the depth of the model in exchange for the performance improvement of the model, without exploring the essential characteristics of the ECG. Previous works based on multi-lead ECG analysis did not pay attention to the correlation among different lead information. The accuracy of the previous works is far from the requirement of technical reliability in the actual clinical environment.

Different lead electrocardiogram is a projection of the vector cardiogram in different directions. The 12-lead ECG can provide a full evaluation of the cardiac electrical activity, and the detection rate of standard 12-lead clinical ECG is significantly higher than single-lead ECG. Single-lead ECG of different diseases has a similar fluctuation trend. For example, in the single-lead ECG signal with normal sinus beats as the main component, the similarity of adjacent beats is high, when arrhythmia or noise disturbance occurs, the similarity of adjacent beats generally decreases. At this time, it is difficult to determine whether it is arrhythmia or low signal-to-noise ratio. In [14], [21], [34]–[41], the input dataset is limited to single-lead ECG records, which provides limited signal compared to a standard 12-lead ECG, and it remains to be determined if those algorithm performance would be similar in 12-lead ECG.

The most commonly used data set currently used to design and evaluate ECG algorithms is the MIT-BIH arrhythmia database [19], which consists of 48 and a half hour 2-lead ECG data records. The beat cycle of the database may belong to the same patient, and different beat data may have the same trend. So the model evaluated using the MIT-BIH-AR database is not robust, and the data sample reported in the MIT-BIH-AR database are insufficient and leads to the overfitting problem.

As the amount of digitized ECG data increases, the performance of deep neural network-based machine learning models can approach and possibly exceed human diagnostic capabilities. After evaluating the largest known CCDD database in the world, MTGBi-LSTM model obtains the most advanced performance on more than 17,000 12-lead ECG records, which proves the effectiveness of our algorithm.

Here, we focused on a central challenge in ECG diagnostics, and proposed the intelligent assistant diagnosis model of CVD based on Res-GCNN and GBI-LSTM hybrid structure. It can accept any multi-lead ECG. We propose a group of Bi-LSTM structures that can Computer-Assisted diagnose ECGs with any number of leads. At the same time, attention mechanism is added to each Bi-LSTM structure to break through the limitation that traditional Bi-LSTM structure relies on internal fixed length vectors when encoding and decoding. The most important thing is that MTGBi-LSTM model explores the characteristic expression of different ECG leads for the first time, which is of great significance for the auxiliary diagnosis of multi-lead ECG.

## IV. Conclusion

In this paper, we propose a Multi-Task Group Bi-LSTM framework to classify various diseases of ECG in order to realize intelligent assistant diagnosis of CVD. This model employs a group Bi-LSTM and Res-GCNN to improve the learning ability of time and space. Furthermore, the model can deeply exploit the features of single-lead and the relationship between each lead of ECG. Attention mechanism and multi-task learning technology are used to enhance the recognition ability of the model, and ultimately to achieve a variety of disease classification. According to the results shown, compared with other classifiers, the F1 of the experimental model in this paper is up to 0.9239 and the results confirm the high accuracy and generalization ability of the proposed model. Experimental results confirm the MTGBi-LSTM is efficient and powerful, and could match state-of-the-art algorithms.

The intelligent assisted diagnosis of electrocardiogram improves the diagnostic efficiency of cardiologists, and we hope that this technology can be combined with online equipment to serve as an early screening tool for cardiovascular disease in areas with limited medical resources.
